# Usefulness of Multi-Parametric MRI for the Investigation of Posterior Cortical Atrophy

**DOI:** 10.1371/journal.pone.0140639

**Published:** 2015-10-19

**Authors:** Andrea Arighi, Mario Rango, Marco Bozzali, Anna M. Pietroboni, Giorgio Fumagalli, Laura Ghezzi, Chiara Fenoglio, Pietro R. Biondetti, Nereo Bresolin, Daniela Galimberti, Elio Scarpini

**Affiliations:** 1 Neurology Unit, Department of Pathophysiology and Transplantation, University of Milan, Foundation IRCCS Ca' Granda Ospedale Maggiore Policlinico, Via Francesco Sforza 35, 20121, Milan, Italy; 2 Magnetic Resonance Spectroscopy Center, Foundation IRCCS Ca' Granda Ospedale Maggiore Policlinico, Via Francesco Sforza 35, 20121, Milan, Italy; 3 Neuorimaging Laboratory, IRCCS Santa Lucia Foundation, Rome, Italy; 4 Department of Radiology, Foundation IRCCS Ca' Granda Ospedale Maggiore Policlinico, Via Francesco Sforza 35, 20121, Milan, Italy; University of Jaén, SPAIN

## Abstract

**Background:**

Posterior Cortical Atrophy (PCA) is a neurodegenerative disease characterized by a progressive decline in selective cognitive functions anatomically referred to occipital, parietal and temporal brain regions, whose diagnosis is rather challenging for clinicians. The aim of this study was to assess, using quantitative Magnetic Resonance Imaging techniques, the pattern of regional grey matter loss and metabolism in individuals with PCA to improve pathophysiological comprehension and diagnostic confidence.

**Methods:**

We enrolled 5 patients with PCA and 5 matched controls who all underwent magnetic resonance imaging (MRI) and spectroscopy (MRS). Patients also underwent neuropsychological and cerebrospinal fluid (CSF) assessments. MRI data were used for unbiased assessment of regional grey matter loss in PCA patients compared to controls. MRS data were obtained from a set of brain regions, including the occipital lobe and the centrum semiovale bilaterally, and the posterior and anterior cingulate.

**Results:**

VBM analysis documented the presence of focal brain atrophy in the occipital lobes and in the posterior parietal and temporal lobes bilaterally but more pronounced on the right hemisphere. MRS revealed, in the occipital lobes and in the posterior cingulate cortex of PCA patients, reduced levels of N-Acetyl Aspartate (NAA, a marker of neurodegeneration) and increased levels of Myo-Inositol (Ins, a glial marker), with no hemispheric lateralization.

**Conclusion:**

The bilateral but asymmetric pattern of regional grey matter loss is consistent with patients’ clinical and neuropsychological features and with previous literature. The MRS findings reveal different stages of neurodegeneration (neuronal loss; gliosis), which coexist and likely precede the occurrence of brain tissue loss, and might represent early biomarkers. In conclusion, this study indicates the potential usefulness of a multi-parametric MRI approach for an early diagnosis and staging of patients with PCA.

## Introduction

Posterior Cortical Atrophy (PCA) is a neurodegenerative disease which is clinically characterized by a progressive and selective decline in visual-processing skills and in other functions localized in the occipital, parietal and temporal brain regions. In contrast, memory and language abilities are relatively preserved, at least at early clinical stages of the disease [1]. PCA is currently considered as a clinical variant of Alzheimer’s disease (AD), due to pathological evidence of neurofibrillary tangles and neuritic plaques deposition as the most frequent findings in the affected regions of the brain [2,3]. Nevertheless, in clinical practice, the diagnosis of PCA is rather challenging due to the unusual clinical onset for AD, the lack of validated diagnostic criteria and awareness among clinicians [4].

Earlier studies based on visual assessment of magnetic resonance (MR) images have consistently reported a bilateral pattern of posterior brain atrophy (i.e., affecting the occipital, parietal and temporal lobes) in cases of PCA [5,6,7,8,9,10,11,12]. More recently, voxel-based-morphometry (VBM), which is an automated and unbiased technique for assessing regional brain volumetrics, has shown a predominant distribution of posterior brain atrophy to the right hemisphere of patients with PCA [13,14]. The intrinsic limitation of volumetric studies is the inability in providing any information about the pathophysiological processes preceding the brain tissue loss. In this perspective, proton MR spectroscopy (1H-MRS) is a noninvasive technique with the ability to provide metabolic information from the brain tissue *in vivo*. Several metabolites of pathophysiological interest can be quantified using 1H-MRS, as demonstrated by its successful application to various forms of degenerative dementia [15,16]. Reduced levels of N-Acetyl-Aspartate (NAA) (a marker of neuronal integrity) and increased levels of myo-Inositol (Ins) (a marker of glial activity) have been identified with specific patterns of anatomical distribution in the brain of patients with typical AD and with frontotemporal dementia [15,16]. So far, to the best of our knowledge, 1H-MRS was used to investigate PCA brains in one previous study only [13]. In that study, Whitwell et al. (2007) focused their investigation on the posterior cingulate gyrus and the precuneus of patients with PCA as compared to patients with typical AD, without reporting any significant metabolic difference between groups [13].

The aim of this study was to assess the regional pattern of grey matter loss and brain metabolites profile in individuals with PCA and to correlate the macrostructural data obtained from VBM to microstructural data obtained from 1H MRS.

## Materials and Methods

### Subjects

Five patients affected by PCA as diagnosed by clinical criteria, were enrolled in the study. Principal patients’ characteristics are summarized in Table 1. The clinical workup included medical history, physical and neurological examination and screening laboratory tests. Additionally, as detailed below and summarized in Table 2, all patients underwent an extensive neuropsychological battery exploring global cognition and all major cognitive domains. According to the clinical characteristics identified by Crutch et al. [4], patients had to fulfill the following criteria: 1) an insidious clinical onset and a gradual progression of symptoms; 2) a cognitive profile dominated by visuoperceptual and visuospatial impairments in the absence of any remarkable deficit of vision itself; 3) a relative preservation of memory and insight; 4) the presence of a complex visual disorder (i.e., elements of Balint’s syndrome or Gerstmann’s syndrome, visual field defects, visual agnosia, environmental disorientation); 5) exclusion of any other alternative diagnosis accounting for patients’ clinical features such as stroke or tumor.

**Table 1:** Principal characteristics of studied patients.

Age	59	70	72	74	78
Sex	M	F	F	M	F
MMSE score (cut-off<24.0)	17.5	15.0	24.0	13.8	16.8
CSF Amyloid β (cut-off> 600pg/ml)	253	375	532	532	528
CSF Total Tau (cut-off< 500pg/ml)	312	983	766	1222	855
CSF Phospho-Tau (cut-off< 61pg/ml)	41	87	89	121	83

Abbreviations: MMSE = Mini Mental State Examination; CSF = cerebro-spinal fluid.

There are summarized here the principal features of all studied patients.

**Table 2:** Neuropsychological assessment of PCA patients.

	Cut-off	Mean (SD)
**Global cognition**		
MMSE [17]	>24.0	17.5 (3.9)
**Visuo-spatial abilities**		
VOSP battery [18]		
Object perception		
Incomplete Letters	>16.0	2.5 (1.7)
Silhouettes	>15.0	5.2 (3.9)
Object Decision	>14.0	9.7 (4.3)
Progressive Silhouettes	>15.0	3.2 (4.7)
Space perception		
Dot counting	>8.0	5.0 (4.1)
Position discrimination	>18.0	12.5 (4.6)
Number location	>7.0	2.0 (1.0)
Cube analysis	>6.0	2.0 (2.0)
**Episodic memory**		
Short story recall test [19]	>4.7	3.2 (2.4)
**Short term memory**		
Digit span [20]	>3.7	3.7 (0.9)
Corsi block tapping task [20]	>3.5	2.3 (2.4)
**Executive functions**		
Phonological word fluency [21]	>17.3	26.7 (4.8)
**Language**		
Naming object [22]	>43.0	28.5 (13.9)
Semantic word fluency [23]	>25.0	19.0 (5.0)

Abbreviations: MMSE = Mini Mental State Examination; VOSP = Visual Object and Spatial Perception

All patients underwent lumbar puncture for CSF quantification by ELISA (Fujurebio, Gent, Belgium) of Beta-Amyloid, Tau and Tau phosphorylated at position 181 (Ptau) proteins.

Five healthy subjects (HS), matched for age and gender with PCA patients, were also enrolled in the study and served as controls. All control subjects underwent a neurological examination to exclude any remarkable impairment.

Moreover, major systemic, psychiatric, and other neurological illnesses were carefully investigated and excluded in all recruited subjects.

The current research study was approved by the Ethics Committee of Foundation IRCCS Ca' Granda Ospedale Maggiore Policlinico, and all procedures followed were in accordance with the ethical standards of the responsible committee on human experimentation (institutional) and with the Helsinki Declaration of 1975 (and as revised in 1983). All subjects (patients and controls) gave written informed consent before entering the study.

### Neuropsychological assessment

The neuropsychological battery used to test the patients with PCA included: Mini Mental State Examination (MMSE) [17]; Visual Object and Spatial Perception (VOSP) battery [18], short story recall test [19], digit span [20], Corsi block tapping task [20], phonological word fluency [21], naming objects [22] and semantic word fluency [23].

For all employed tests, we used the Italian normative data for score adjustment (age, gender, and education) and for the definition of cut-off scores of normality (determined as the lower limit of the 95% tolerance interval for a confidence level of 95%). For each test, normative data are reported in the corresponding references.

### Conventional MRI and volumetric brain acquisitions

All MRI data were acquired at 1.5 T (Siemens Avanto, Erlangen, Germany) equipped with an head matrix coil. 3D magnetization-prepared rapid gradient echo (MPRAGE) T1-weighted scan (TR 2300 ms, TE 2.84 ms, in-plane field of view [FOV] 512 x 512 mm2, slice thickness 1 mm, flip angle 12°) was acquired from all recruited subjects. T1-weighted images were visually inspected for a qualitative assessment of macroscopic atrophy, and to check for the quality of data before carrying out a quantitative volumetric analysis. Additionally, in all subjects, a T2-weighted scan (TR 5750 ms, TE 108 ms, field of view 280 x 320, slice thickness 5 mm) was collected to exclude the presence of any remarkable macroscopic brain abnormality.

### Voxel-based morphometry

MRI data were processed using an optimized VBM protocol in Statistical Parametric Mapping 8 (SPM8; Wellcome Department of Imaging Neuroscience; www.fil.ion.ucl.ac.uk/spm/). Briefly, for each subject, segmentation and normalization produced a GM probability map [24] in Montreal Neurological Institute (MNI) coordinates. To compensate for compression or expansion during warping of images to match the template, GM maps were modulated by multiplying the intensity of each voxel by the local value derived from the deformation field [25]. All data were then smoothed using a 12-mm full width half maximum (FWHM) Gaussian kernel. Modulated and smoothed GM maps were analyzed in SPM8, using a sample t-test for the comparison between patients and controls and a regression model for assessing possible associations between patients’ regional grey matter volumes and CSF levels of tau (marker of neurodegeneration). Total intracranial volume (ICV), age and gender were entered as covariates of no interest. For every T-contrast, we applied the family wise error (FWE) correction for multiple comparisons, and we accepted as significant p values of less than 0.05 at cluster level.

### Single Voxel 1H MRS

**Fig 1:**
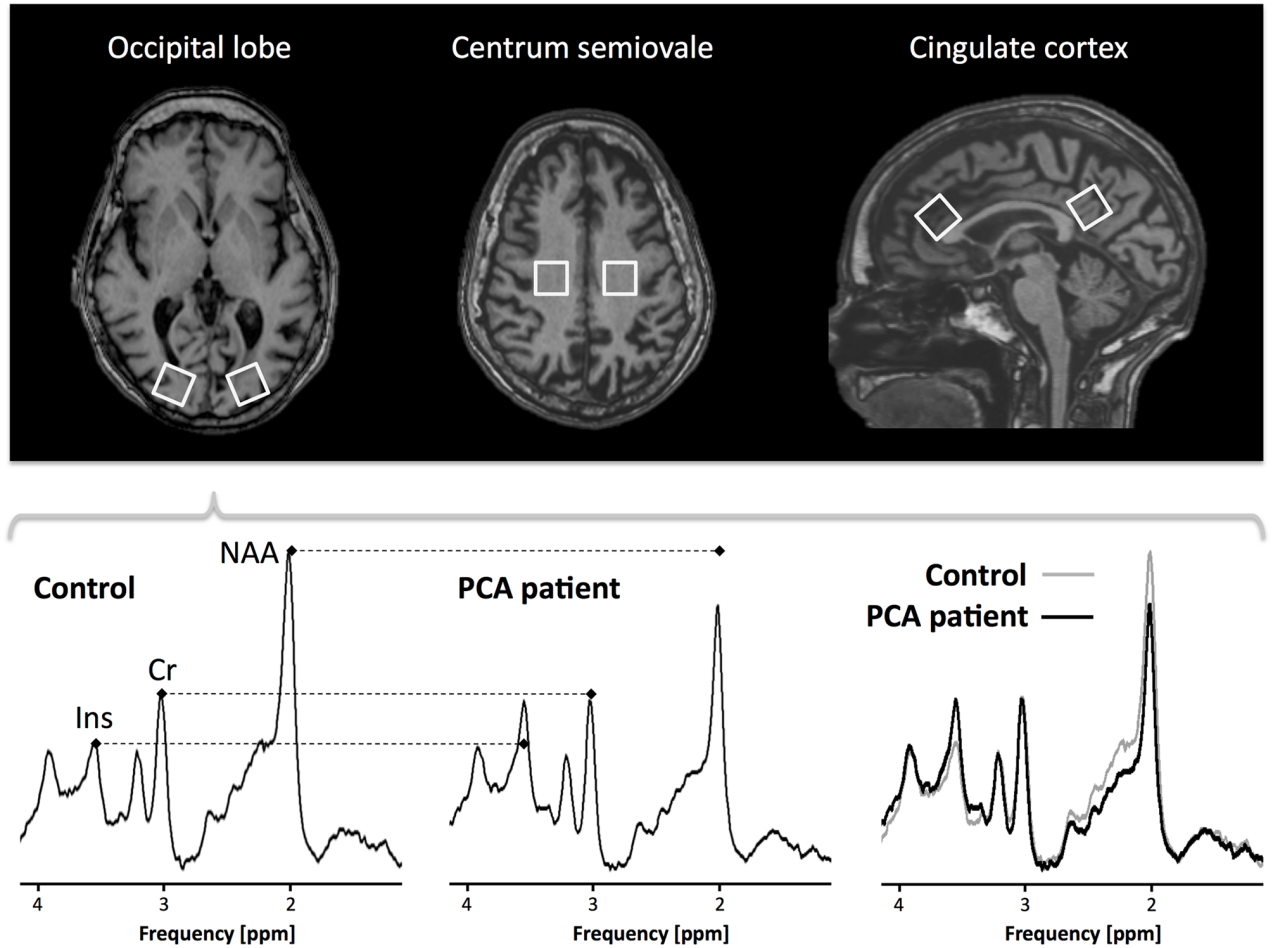
Volumes of interest (VOIs; size = 20x20x20mm) investigated by 1H MRS (top panel). Typical spectra obtained from VOIs positioned in the occipital lobes of a control subject and a patient with PCA are also shown (bottom panel) See text for further details.

In a dedicated session, all subjects underwent 1H-MRS. 1H-MR spectra were acquired without and with partial water suppression, from the following 20x20x20mm volumes of interest (VOIs): 1) VOI 1 and 2, aligned along the calcarine fissure and located within the white matter of each occipital lobe (right and left); 2) VOI 3 and 4, located in the posterior and anterior cingulate cortex; 3) VOI 5 and 6, located within the centrum semiovale of the right and left hemisphere (See Fig 1). Data were obtained under baseline conditions, with the participants lying in a diminished light, asking them to keep their eyes closed. A point resolved spectroscopy sequence (PRESS) was employed for spectra collection, setting the following acquisition parameters: TR 4000 ms, TE 30 ms, 1024 points per spectrum, 32 acquisitions in partial water suppression spectra and 8 acquisitions in water suppression spectra.

Spectral analysis was performed, and data quantified, using the Linear Combination of Model spectra (LCModel, http://s-provencher.com/pages/lcmodel.shtml) method and software [26]. In each VOI, the metabolite groups quantified with a good signal-to-noise ratio (SNR) were: the N-acetyl aspartate (NAA), Creatine (Cr), Choline (Cho) and myo-Inositol (Ins). As previously described [13,27], the following metabolite ratios were calculated from each spectrum, and used for between-group comparisons: NAA/Cr, Ins/Cr, Cho/Cr. Finally, to reduce inter-subject variability and minimize the risk of type II errors, for each bilateral VOI (i.e., VOIs 1 and 2, and VOIs 5 and 6) metabolite ratios were averaged. Two-tailed unpaired Student’s t-tests were used for between group comparisons, with statistical threshold set to p values <0.012 after Bonferroni’s correction for multiple comparisons.

## Results

### Subject Characteristics

Principal characteristics of the studied group of PCA patients are summarized in Table 1. There were no significant differences between patients with PCA and HS with respect to age (73.2±7.2 and 68.8±9.8 years respectively) and gender distribution (M/F:2/3 and 3/2 respectively). All patients showed similar symptoms at onset, including visual, reading and driving difficulties, dyscalculia, spatial disorientation and recent memory disturbances. On neurological examination, elements of Balint's and Gerstmann's syndromes were documented in all patients. Neuropsychological testing revealed a remarkable impairment in global cognition in all patients, as measured by MMSE (Tables 1 and 2). With respect to assessment of single domains (Table 2), patients performed poorly in most areas of cognition, with their worst performance in visuospatial abilities. With respect to episodic memory, on average, patients performed under the cut-off of normality. However, this performance was higher than that expected in patients with typical AD and a similar level of global cognitive impairment [28]. CSF analysis showed decreased beta amyloid levels in all patients and increase of Tau and Ptau concentration in 4 out of 5 patients (Table 1).

### Voxel Based Morphometry

**Fig 2:**
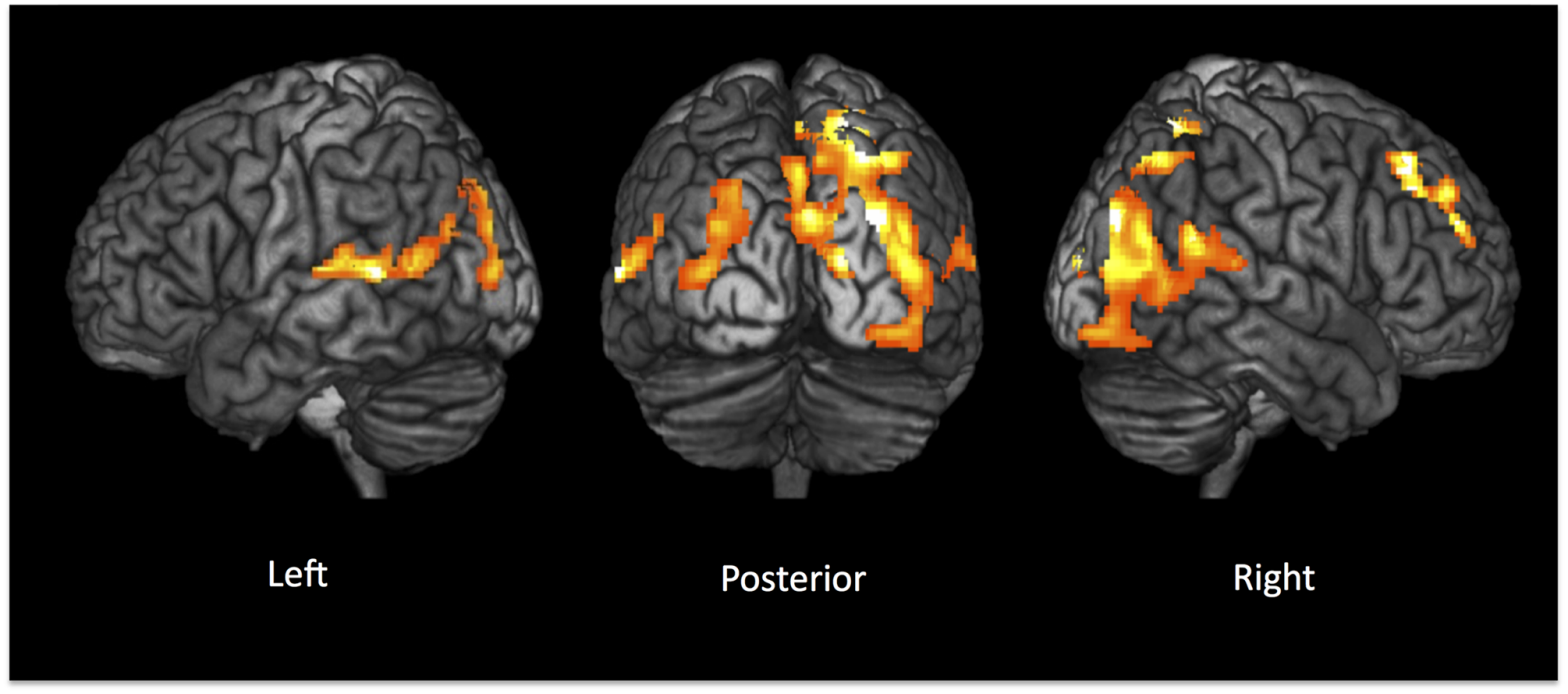
VBM analysis: PCA patients compared to healthy controls showed a widespread pattern of bilateral grey matter loss, predominantly affecting the posterior areas of the brain. The opposite contrast (PCA patients more than controls) did not reveal any significant difference. Statistical threshold: p<0.05 Family Wise Error (FWE) corrected at cluster level. See text for further details.

When comparing PCA patients against controls, the former group showed a widespread distribution of regional grey matter loss affecting the posterior brain areas (predominantly on the right hemisphere) (P_FWE_corr<0.05), as shown in Fig 2. This pattern of grey matter atrophy included the parastriate and peristriate occipital lobe cortices (BA18 and BA19 respectively) and the posterior part of the temporal lobes (superior temporal gyrus BA22, BA41) and of parietal lobes (angular gyrus BA39, precuneus and superior parietal lobule BA7). Additionally, a region of significant grey matter loss was also identified in the right frontal lobe of PCA patients (frontal eye field BA8, anterior prefrontal cortex BA10). No regions of reduced grey matter volumes were found in healthy controls as compared to patients. No significant correlation was observed between patients’ regional grey matter volumes and CSF tau levels.

### 1H MRS

**Fig 3:**
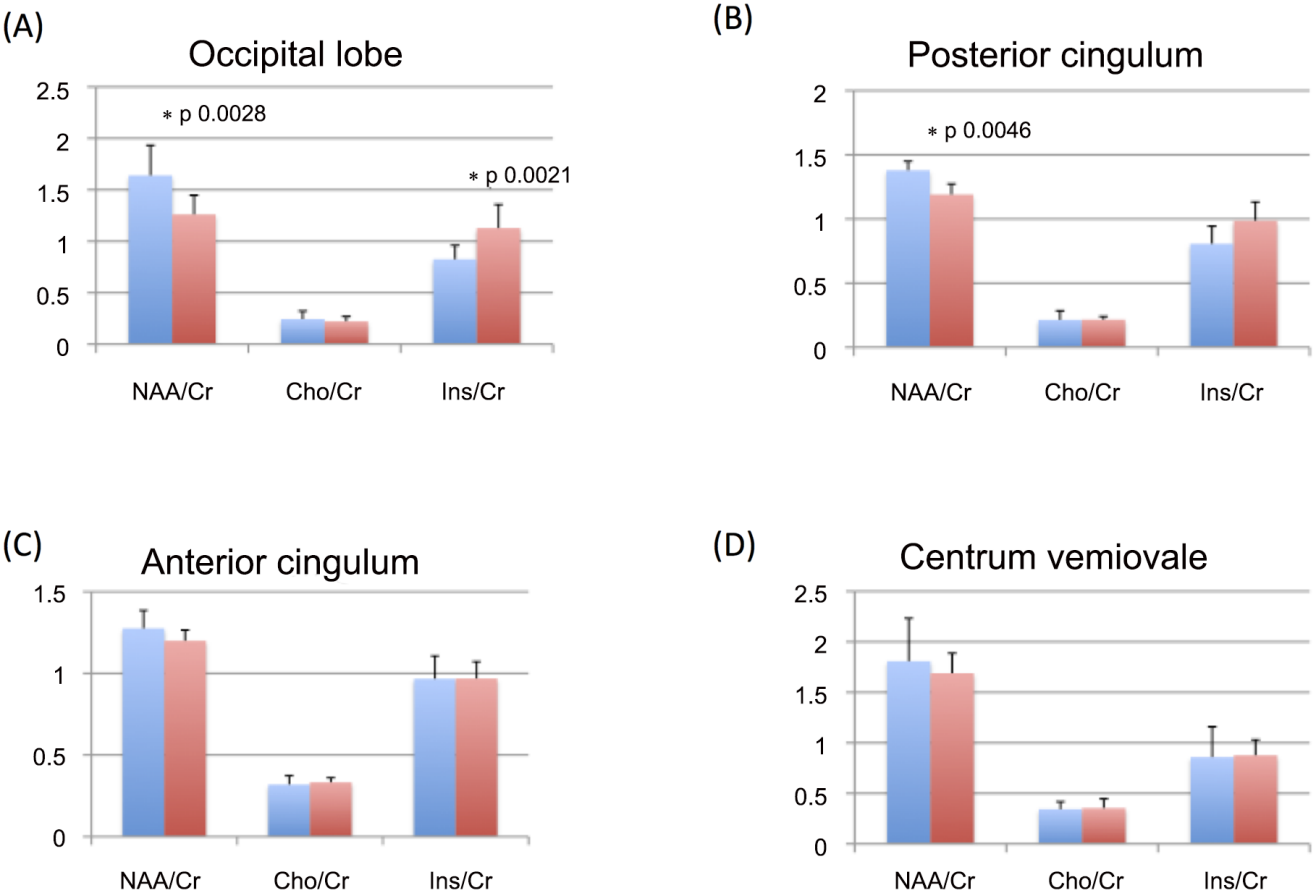
Column graphs showing mean and standard deviations of 1H MRS metabolite ratios (NAA/Cr, Cho/Cr and Ins/Cr) form the different VOIs in patients with PCA (orange) and healthy controls (blue). In the case of bilateral VOIs (i.e., occipital lobe and centrum semiovale), left and right measures were averaged. PCA patients compared to controls showed a significant decrease of NAA/Cr in the occipital lobes (panel A) and in the posterior cingulate gyrus (panel B). Additionally, PCA patients showed a significant increase of Ins/Cr in the occipital lobes (panel A). No significant between group differences were observed in the anterior cingulum (panel C) and in the centrum semiovale. Statistical threshold: p>0.012 after Bonferroni’s correction. See text for further details.

The averages of the metabolite ratios obtained from all voxels positioned in the occipital lobe (right and left) showed decreased neuronal integrity marker NAA/Cr (PCA 1.26 ± 0.19, HC 1.64 ± 0.29, t-test p 0.0028) and increased glial marker Ins/Cr (PCA 1.13 ± 0.23, HC 0.83 ± 0.14, t-test p 0.0021). Voxels from the posterior cingulate cortex revealed a decrease in patient’s NAA/Cr (PCA 1.19 ± 0.08, HC 1.38 ± 0.07, t-test p 0.0046) (Fig 3). When comparing right and left hemispheres in the patient group, there was no significant difference in any of the considered VOIs (p values ranging from 0.25 to 0.98).

## Discussion

Herein, we described a group of patients with PCA, characterized clinically and neuropsychologically, who were investigated using quantitative MR techniques. This clinical condition has become of increasing interest, since the clinical onset of a relevant proportion of patients with Alzheimer’s disease is dominated by non-typical symptoms [29]. The clinical features of our PCA patients are similar to those previously described by others [5,9,11,30,31]. Their profile was characterized by visual, reading and driving difficulties, dyscalculia, spatial disorientation, and elements of Balint's and Gerstmann's syndrome. Less severe memory impairments for recent events were also present in our patient group. A diagnosis of amyloid-based dementia was supported by CSF biomarkers, with the demonstration of low levels of beta-amyloid in all cases, and increased levels of total Tau and Ptau the majority of them. This CSF profile has been associated with the presence of Alzheimer's disease pathology (i.e., neurofibrillary tangles and amyloid plaques,) with a predictive value of 90% [32], and PCA is currently regarded, in most cases, as a focal variant of Alzheimer’s disease [29,31,33,34,35].

When using VBM for an unbiased assessment of grey matter volumes, our PCA patients showed a posterior pattern of cortical atrophy, which fits well with their clinical and neuropsychological characteristics. The anatomical distribution of GM loss in the patient group involved the secondary visual cortex, the visual association cortices and the posterior part of the temporal and of parietal lobes. All these cortical regions are known to play a major role in visual perception and interpretation of sensory information, and their selective damage has been consistently reported in patients with PCA [9,36,37,38]. Our PCA patients showed also an asymmetrical distribution of GM atrophy, which was predominant on the right hemisphere. This finding is also consistent with previous reports, which have demonstrated right hemisphere predominance in patients with PCA [8,10,12,13,39,40]. Additionally, our PCA group revealed an area of focal GM loss in the right prefrontal cortex. This area coincides with the frontal eye fields, which have been previously described as hypomethabolic and atrophic in PCA patients [10,13]. This focal loss of GM may also contribute in accounting for some peculiar clinical features of PCA such as ocular apraxia, and is likely secondary to structural disconnection of fibers originating from the occipito–parietal cortices. Additionally, our specific group of patients showed some minor deficits in memory retrieval and executive functions, which might be, at least partially, explained by prefrontal damage. Regarding CSF tau levels, which represent a marker of neurodegeneration, there were no significant correlations between patients’ grey matter volumes and such levels. Nevertheless, four out of five patients had very high tau levels (clearly out of reference range) with poor intersubject variability. The fact that values were very similar across patients, together with the small sample size, may account for the lack of correlation.

GM volumetrics is certainly quite a useful approach to associate clinical aspects of disease with anatomical patterns of brain tissue damage. However, atrophy is only the final step of neurodegeneration, and its assessment is poorly informative on the underlying pathophysiological mechanisms. Conversely, 1H MRS provides metabolic information on the brain tissue *in vivo*, and has already been used to investigate single regions of PCA brains [13]. Notably, this is the first study that applied 1H MRS to multiple brain regions in the same cohort of PCA patients, thus allowing, together with GM volumetrics, a better definition of the pathophysiological trajectory of the disease. Indeed, within the limitation of a cross-sectional design, metabolic abnormalities are supposed to precede the brain tissue loss, although this speculation needs to be confirmed by longitudinal investigations. When focusing on the occipital lobes, 1H-MRS revealed decreased levels of NAA/Cr and increased levels of Ins/Cr in PCA patients compared to controls. NAA is a neuronal protein, whose decrease is widely regarded as a marker of neuronal loss [27], especially in neurodegenerative conditions. Conversely, Ins is considered as a marker of gliosis, which we found to be altered in the occipital lobes (the anatomical core of PCA pathology), but not in other brain areas. Changes in both markers are likely to occur at different disease stages and in different brain regions, and might represent sensitive biomarkers of disease evolution. However, longitudinal studies are needed to clarify the usefulness of MRS in providing early disease markers and information of potential prognostic value. Consistent with a previous report [13], we observed here a reduction of NAA/Cr also in the posterior cingulate cortex of our PCA patients. This is of particular interest for at least two reasons. The posterior cingulate cortex is the most critical node of the so-called default mode network, which goes disrupted across AD progression [41] but not in other forms of dementia, such as, for instance, the frontotemporal dementia [42]. This supports the idea that PCA is in most cases a focal variant of AD. Second, this selective damage might contribute in accounting for the memory deficits we observed in our PCA patients, which were present but less pronounced than those expected in patients with typical AD [28].

The centrum semiovale and the anterior cingulate cortex of our PCA patients did not reveal any significant abnormality when considering either structural or metabolic measures. These negative findings are also of clinical interest, as they suggest a potential role of quantitative MRI in supporting the differential diagnosis between PCA and other neurodegenerative forms of cognitive decline. However, again, further studies on larger populations are needed to address this point.

A major limitation of the present study (and previous research in the field of PCA) is the relatively small number of participants. In an attempt to overcome small samples and other issues of individual studies, Alves et al. in 2013 performed a meta-analytic review of neuropsychological and brain morphometry PCA studies [14]. With respect to the common data between such a meta-analysis and the current investigation, results are fully consistent. However, due to the relatively low incidence of PCA, future multi-centric studies (possibly with a longitudinal design) employing comparable protocols of data collection are needed. This is relevant not only for increasing our knowledge on PCA pathophysiology, but also for planning future clinical trials. With respect to proton MRS, according to our data, it may be useful for a better definition of the disease, and could also be considered in view of future treatments for a more accurate patient selection and monitoring.

Due to the single-voxel MRS approach we employed here, another limitation of the current study is the lack of metabolic information from the whole brain. In particular, it has been recently shown that the parietal lobe is an early target of PCA pathology [43,44]. Again, future MRS studies are needed to further explore this aspect.

In conclusion, within the limitation of a small sample size, this study indicates the potential usefulness of a multi-parametric MRI approach, in combination with CSF biomarkers, for the differential diagnosis and staging of patients with PCA. This is relevant not only for speculative reasons but also for setting up reliable tools of disease monitoring to be applied to studies of natural history and clinical trials.
